# The social representation of the identity perception among China’s new social strata: a central nucleus analysis

**DOI:** 10.3389/fpsyg.2025.1722048

**Published:** 2026-01-22

**Authors:** Li-Ju Li, Fang Luo, Wei Zong, Fa-Wen Hu, Wen-Zhi Liao, Yu Ding

**Affiliations:** 1 College of Education, Honghe University, Mengzi, Yunnan, China; 2 Centre for Studies of the Ethnic Minorities in the Borderlands of Southwest China, Yunnan University, Kunming, Yunnan, China; 3 School of Building Information, Guangdong Construction Polytechnic, Qingyuan, Guangdong, China; 4 College of Applied Technology, Yunnan Minzu University, Kunming, China

**Keywords:** central nuclear, identification, K-core analysis, new social class, social representative

## Abstract

**Introduction:**

China’s new social strata represent a critical demographic for social governance, with shared identity perceptions serving as a cohesive force for group solidarity and behavioral regulation.

**Methods:**

This study employed a word association task with 256 participants from China’s new social strata, followed by lexical network analysis including K-core decomposition to uncover hierarchical identity structures.

**Results:**

The social representations of “identity” among this demographic comprise a primary component and three K-core sub-networks. The central core identity system contains 37 pivotal elements classified into categorical identity, relational identity, and symbolic identity, with “Status” and “ID Card” as the most central elements.

**Discussion:**

The new social strata perceive identity across three interconnected dimensions forming a “periphery-margin-core” tripartite network model. This structure reveals how institutional mechanisms, particularly China’s household registration system, shape subjective identity perceptions in contemporary society.

## 1 Introduction

The new social strata, a product of China’s identity and household registration-based societal classification, primarily refer to the middle-income groups engaged in management and technical work within non-state-owned enterprises and social organizations under the current socio-economic system. Occupationally, this group includes management and technical personnel in private and foreign-invested enterprises, as well as freelancers and new media practitioners in emerging economic sectors and industrial models (Zhang and Yang, 2022; Zhang and Yuan, 2022). The diversity within this group leads to varying interest demands, value orientations, social norms, and class positions in economic life, social interactions, and professional behaviors. This diversity hinders the achievement of a unified identity consciousness and narrative identity within the group, leading to identity crises and difficulties in identity construction. In fact, amidst China’s strategic layout for comprehensively deepening reforms, the new social strata have continued to expand, grow in economic strength, and exert greater social influence, becoming builders, a vital force, successors, and pillars of the cause of socialism with Chinese characteristics (Jiang et al., 2023). Compared to the extensive discussions based on externally imposed identities, revealing the intrinsic identity consensus within the new social strata may play a more significant role in unifying thoughts, fostering identification, and uniting the group. Due to this, a psychologically meaningful identity, aligned with the group’s prevailing identity, can thus be constructed, one which serves as its representative image in society as well.

Identity exists across various levels, from psychological to social and political, which means we need to look at it on different planes. Following the perspectives given by Skeggs (2004) and Bourdieu (1984), we take identity as symbolic in essence with a cognitive representational function, which means we use it as a social position and social recognition. At an individual level, identity shows self-categorization by adopting roles or groups, through which the association between self and roles can be formed (Wen, 2017). At the collective level, identity is a boundary-making process through which groups differentiate themselves and claim legitimacy (Lamont and Molnár, 2002), which in China is further complicated by the active role of the state in identifying and classifying people through hukou systems and occupational categorization (Yan, 2010). In this institutional context (Whyte, 2010), state-mediated identity classifications interact with identity aspirations on an individual level, especially those of groups that fall into ambiguous categories in the socialist market economy, such as the new social strata in China (Li and Zhao, 2017). In this study, we define “identity perception” as the cognitive conception of identity in the conceptual lexicon of new social strata members, distinguished from “identity categorization” (social positioning processes) and “identity construction” (both individual and state-mediated identity formation processes), where the latter two pertain to the social and political dimensions of identity. But the concept of identity can also convey both an individual’s social status and his or her self-validation regarding what his or her role in society entails. To address the questions “Who am I?” and “How do I see myself?” in line with social identity theory (Tajfel and Turner, 1979), individuals largely derive meaning and self-definition through the groups they are identified with, thus placing value on groups they both choose and are allocated into. Through social categorization and self-categorization processes, we psychologically distinguish “us” from “them,” resulting in different cognitions, emotions, attitudes and behaviors toward insiders and outsiders. The group’s size, capacity and hierarchy depend on the criteria used for classification. Therefore, an individual can belong to many groups simultaneously or hold several different identities within one group. Identity is thus neither unitary nor singular. Instead, individuals embody many social identities. Individuals can have multiple intersecting social identities that form through multiplicity, diversity, and complexity along various dimensions. Identities interact and overlap to form a multidimensional identity structure (Zhang and Xu, 2023). They also contain a hierarchical system that includes core and peripheral identities. In an ever-changing professional situation, the specific characteristics that different social categories of the new strata display when they engage in social interaction reflect various pieces of social information as well as symbols, based on which these characteristics are to be defined. Ismail et al. (2025b) demonstrate how media narratives in Indonesia mediate between global influences and local values when constructing national identity through sports naturalization policies. In fact, different dimensions of individual identity may manifest themselves differently within diverse social domains (gender, age, ethnicity, occupation, class, political affiliations, hometowns and domicile) when people come to acquire an identity relating to the distinct reference groups found within them. When we have understood their respective identity perspectives of the new social strata, this deeper understanding will help us find those common core identity elements that they share, from which we can try to infer what the people they collectively embody hold as their social normative values, beliefs and ideologies, or their ways of representing those things.

Indeed, people have several identities during identity formation because of their relational ecology and role commitment. These identities are formed through both social construction by others and self-construction. Individuals locate themselves within the complex web of social relationships, and new social strata play an important role in the system (Yu and Li, 2022). Drawing from their work expertise and professional status, they are able to navigate through these intersections between them. Besides their participation in traditional structures and workplace relationships, they gain access to numerous social, economic, cultural, and organizational resources, which enable them to pursue varied goals and self-identification through multiple occupational organizations. In terms of occupation moving pattern, however, they occupy positions mainly in those private economic organizations and social groups, with the characteristics of mobility toward outside society, younger age, and short service period. Such types of physical migration can also result in an identity crisis and anxiety for migrants (Qin, 2005). The new social strata that are constantly moving need to reevaluate themselves frequently under the brand-new working environment and social relations and change their identity system in a new way (Yuan et al., 2023). This complex identity formation process reflects broader global trends in identity research, which Ismail et al. (2025a,b) identify as increasingly interdisciplinary and focused on the intersection of psychological, sociocultural, and political dimensions. Its fresh self-concept and identities change the self-perception. What is needed is a clear understanding of the new social group’s actions, thoughts, emotions, and their relationships with each other. Essentially, the social mobility they experience leads them to redefine themselves, re-evaluate their position in society, and think about and rethink who they are, thus resulting in changes in mental perspectives on the self. Social mobility causes identity concerns and identity conflicts; the formation and transformation of identity form the critical problems of the emerging social strata. Therefore, studying the social representation of the identity structure can help analyze the identity structure of the emerging social strata and further comprehend its identity cores.

As entities at odds with the organizational structure, people in the new social strata differ from traditional social groups in terms of origin, composition, movement, lifestyle and so on (Feng and Chen, 2022). They have a duality of characters; that is to say, they are not only characterized by social class but also by the institution, and there are some differences in their expression on the level of both the government and academia. Generally speaking, the government mainly adopts strategies for spreading messages and truth-telling, removing the origin of the needs of national governance and united front work. It is easier to give the collective identity of “non-public economic personages” due to the nature of the economy and occupation structure, and asserting their identity affiliation is “socialist laborer” or “socialist builder.” The above classification can be divided into four employment and professional types: Management and technical staff in private industry enterprises, foreign-invested enterprises, people engaged in intermediary and social organizations, freelancers, and people in new media represent different groups with unique occupational attributes. The inclusive occupation narrative strategy is adopted to assist their shaping of social belonging and a collective identity among the group, while the academic narrative approach highlights and encapsulates group characteristics and occupation features of new social strata, outlining their class-based identity traits such as “young age, concentration in megacities, high educational attainment, high income, positive political and social attitudes, significant public opinion influence, active presence in cyberspace, and strong willingness to participate in public affairs” (Lu and Zhou, 2022). Academics often classify them as the middle class or intermediate strata (Zhang and Yang, 2022). Clearly, these two identity narrative strategies primarily attend to the scope and characteristic identities of the new social strata, with notable differences in purpose and focus. Despite these two identity attribution mechanisms, both represent an other-constructed perspective on understanding the identities of the new social strata, emphasizing the imposed and external understanding of their identities. They pay less attention to how members of this group perceive and understand these identities themselves, lacking revelation of the content of their identity representations and their organizational patterns.

From an epistemological perspective, identity, as a unique form of social representation, influences human cognition, decision-making, and behavior, mediating the relationship between individuals and social realities (Chryssochoou, 2003). Its consistency between subjective confirmation and recognition by others is the result of social representation in social interactions and orderly communication among specific groups. As a methodology for constructing social consensus and gaining insights into knowledge systems, social representation is both a means for community members to collectively construct specific social objects and their social meanings and a system of values, ideas, and practices that establishes social order and facilitates communication (Sammut et al., 2015). Methodologically, social representation has become a primary approach for studying the common sense or meaning generation of diverse social and cultural groups (Sammut et al., 2015). Accordingly, based on the central nucleus theory (Giumelli, 1993; Molinari and Emiliani, 1996), it is possible to postulate that the representation of social identity consists of two cognitive systems: a central one and a peripheral one, each of them possibly incorporating several different contents forming the center and periphery, respectively, among which the center component is relevant, defining the meaning of social representations as the central element shapes individuals’ perceptions and guides their actions (Wachelke, 2008). While the periphery includes beliefs, ideas, and stereotypes contextualized to particular situations, which can be modified to fit the evolving characteristics of society and social relationships (Abric, 2001). To verify whether this identity-representation pattern hypothesis is correct or not, this study draws on the previous social representation research of “pain” (Lin et al., 2013), which utilizes “identity” as the stimulus word to construct a lexical association network of the social representation of “identity,” and adopts Cao et al.’s (2012) method of applying network technologies to represent conceptual knowledge about identity in order to explore the constituent parts and internal organizational pattern structure of the social representation of identity amongst the new social stratum people, with the aim of ultimately providing certain theoretical guidance and real-world references for their construction of identity and promotion of psychological identity development.

## 2 Materials and methods

### 2.1 Survey participants

In this research, 256 participants of the NSS were surveyed, the data were collected using convenience sampling, and the target group members were identified based on the open call for survey participation at “Wenjuanwang,” which distributed its QR code to friends and colleagues through WeChat professional networks and other recruitment strategies. Even so, this sampling is not fully representative of the broad and dispersed population of the NSS. Geographically speaking, it has a limited effect. Geographically, they lived in Tier-1 Cities (48.3%), Tier-2 Cities (36.7%) and Tier-3 or smaller Cities (15.0%). The monthly income breakdown was as follows: less than RMB 8,000 (23.8%), RMB 8,000-RMB 15,000 (41.2%), RMB 15,000-RMB 30,000 (27.4%), and more than RMB 30,000 (7.6%). This is similar to previous demographic studies on China’s new social strata’s distribution (Zhang and Yang, 2022; Lu and Zhou, 2022); however, the significant regional concentration remains prominent in the eastern provinces. The age of the respondents ranged from 18 to 55 years, with an average age of 30.35 years (SD = 8.67). The gender distribution was relatively balanced, with 148 males and 108 females participating in the survey. Educational backgrounds varied among the participants, with 22 holding graduate degrees, 97 possessing bachelor’s degrees, 114 having college diplomas, 18 with high school/secondary vocational/technical school qualifications, and 5 indicating others, reflecting the high diversity in levels of education for these new social strata; and in terms of occupation, the participants in this study covered most categories in the intermediate organizations outside the SOEs. In occupation distribution: there were 19 people working in service intermediary organizations, including employment, advertising, public relations, real estate, 11 of them were either individual business owners or contractors; there were 10 from intermediary organizations, including different types such as lawyer, accountant, appraiser, securitization, and arbitration organizations; there were 48 managers who worked for private enterprises, and 35 technicians from the private enterprises; there were also 21 people working for social organizations, 15 people being managers from private enterprises, 19 being technicians from private enterprises, 4 managers from foreign-funded enterprises, 4 technicians from foreign-funded enterprises, 24 working in the new media, and 3 employees from industry-specific intermediary organizations (such as industry association, society, chamber of commerce and research institution) and 43 freelancers.

A fully inclusive sampling design is needed to capture a representative social snapshot of the nascent social stratum and establish an adequate framework of reference for examining their identity constructs as well as the social representations that drive their professional and social identities.

### 2.2 Stimulus materials

To select the 13 stimulus words, we used a systematic 3-stage process: first, we conducted semi-structured interviews with 30 people from the target population; second, we extracted frequently occurring identity terms from China’s new social strata documents and academic literature using thematic analysis techniques; and finally, three social psychologists and two sociology scholars reviewed and refined the candidate list, ensuring both ecological validity and theoretical relevance. The final set includes “identity,” “builder,” “new social strata,” “Chinese,” “democracy,” “harmony,” “equality,” “justice,” “patriotism,” “integrity,” “politics,” “security,” and “interests”. While terms like “harmony,” “patriotism,” and “builder” reflect China’s political discourse, they were included because preliminary interviews revealed these concepts frequently appear in participants’ spontaneous discussions about social identity. We acknowledge that such politically charged terms may trigger social desirability bias, a limitation addressed in the “4 Discussion” section.

Before the analysis, we did a large amount of linguistic preparation work. (1) Synonym merging used terms with identical meanings as defined in HowNet (Dong and Dong, 2006), such as “status” and “position.” (2) Frequency filtering: Responses that appeared less than three times were removed to decrease noise. (3) For polysemous terms (e.g., interest), context-based disambiguation was conducted by three bilingual coders. (4) Translation validation: All Chinese responses were translated into English by two different translators, and any differences were ironed out via a group discussion to agree on one form of text.

The terms were put together in the “Word Association Task” questionnaire presented via Wenjuanwang, an online form-making platform. The platform is very convenient to use, has many uses, and has allowed us to send out and collect the survey questionnaires through such an easy method. People were encouraged to give answers spontaneously and write down three words that came to mind for each word they saw as the stimulus word. This experiment designs a way to tap into individuals’ spontaneously generated cognitive associations with the concepts of identity, as well as social constructs associated with it. In order to eliminate the impact of order effects, the presentation order of the stimulus words is random for every subject. The sequence of the stimulus words has been randomized using Wenjuanwang’s automatically shuffled questions function. Words varied from one participant to another and were neither fixed nor limited by any ordering or sequencing, offering complete freedom in the subject’s choice of words to describe their own position on the social ladder. By using this research methodology, researchers can limit the occurrence of bias that may result from a certain format of questionnaire sequencing. Relying on the use of the Randomized Word Association Task (RAWAT), a deeper understanding of the complex social representations of identity formed among individuals belonging to the new social stratum is possible, and the associations produced by the respondents allow further analysis of what central/peripheral elements form part of their identity space, while also allowing an insight into the social hierarchies created as a result of the arrangement of salient domains.

### 2.3 Survey procedure

Start off through the WeChat platform, so you can just entrust your relatives and friends to use their own networking potential in terms of different professional spheres or even professional associations, chambers of commerce or even work-related groups for distributing either the link or the QR code poster from the “Word Association Questionnaire.” Subsequently, we ask that those who participate in the questionnaire complete it on their own, following the instructions below. For confirmation purposes, the project group name and number will be given: the National Natural Science Foundation of China Project Group 72061012. Below is the translation of specific instructions given above, assuming different roles.

*Hello! Thank you so much for finding time to do the questionnaires. The purpose of this survey is to gain insight into how people at different social levels in China perceive their own identities, characteristics, and relationships, and how these factors help with understanding China’s society. Also, the data can be used as both an indicator for social construction and as some raw material on which the policymakers of class governance can rely. The task is a word association task. When you see these 13 words (they are also referred to as stimulus words), I hope you can focus on writing down what appears in your head after seeing each word—whether it’s a person, an event, or an object, or even another word, sentence, or short phrase. Write down just 3 associations that pop into your mind after viewing each word. There is no correct response, and each one of them is of equal value. We assure you that your responses will not be traced back to individuals or organizations. They will never undergo any statistical analyses; instead, we will utilize network analyses to find common problems and recommend corresponding interventions. Thank you very much for your support! Let’s begin our experiment!*

*Note: After finishing this, you will be invited to participate in a lucky draw for cash red envelopes with various amounts. It’s possible that you win, even though we don’t expect it. If you get a red envelope, we hope that you will enjoy it. We truly hope that everything in life goes as you wish!*

Lastly, upon completion of the word association task, participants are required to provide their personal information, including gender, age, highest educational attainment and major, occupation type, professional role, position or title, current social identity, the name and address of their employer, and the industry they are currently working in. Additionally, participants are asked to rate their agreement with two identity statements on a 5-point scale: “Are you a contributor to the cause of socialism with Chinese characteristics?” and “Do you consider yourself a member of China’s new social strata?” Higher scores indicate stronger identity recognition.

### 2.4 Vocabulary network analysis

Using SPSS 22.0 for Windows, a chi-square test was carried out on the network indicators of identity social representations; using Pajek 5.06 for Windows to analyze the social representation structure of identity, as well as the visualization network graphs thereof. The network properties are measured by the above indicators (Newman, 2001; Wouter, 2005).

#### 2.4.1 Construction of stimulus word network

**FIGURE 1:**
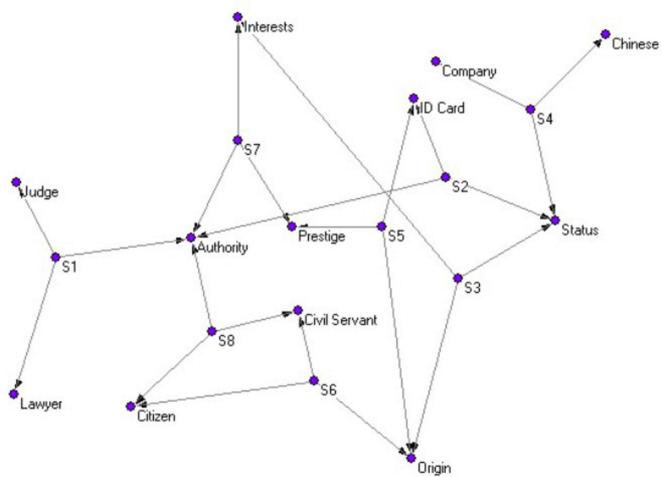
“Subject → response” bipartite network.

**FIGURE 2:**
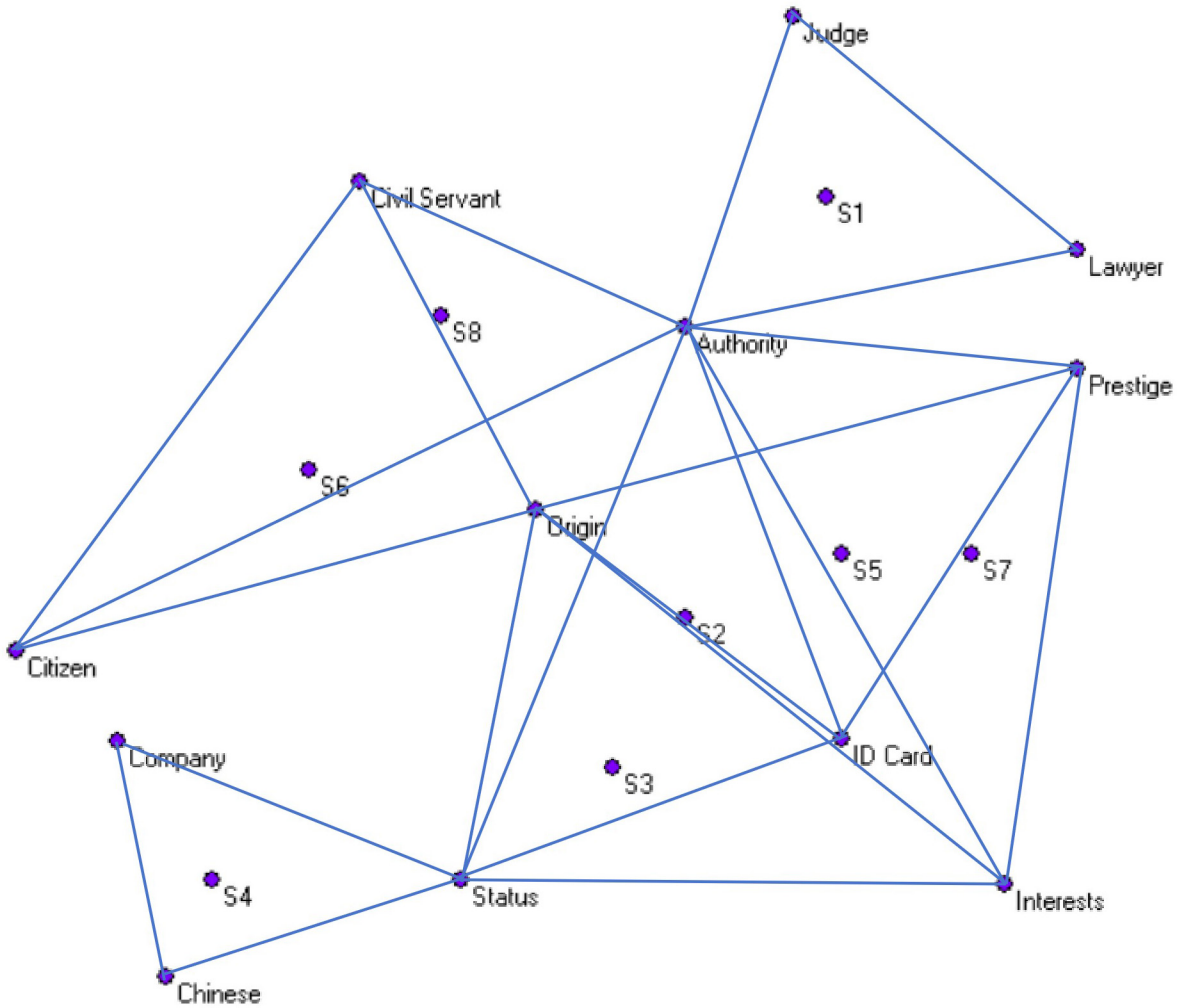
“Response-response” network.

Drawing on the network perspective, the evoked words and their associated links to other items are regarded as network nodes and links, respectively. Following this, the undirected evoked word networks were constructed based on the procedure (Lin et al., 2013). This means that the present work firstly used participant-evoked words to create an undirected evoked word network, which we ultimately applied in this study to elucidate the social representation network underlying “identity perceptions” of the new social strata. The procedures in constructing these social representations are described as follows: First, both the participants and the vocabulary they elicited in response are regarded as different nodes within a network, constructing a two-mode network reflecting the one-way connection between the participants and their responses, as depicted in Figure 1. In this network, the three response words generated by each participant’s association in the context of the stimulus word “identity” exhibit co-occurrence relationships, forming a strong cognitive connection. Secondly, based on the co-occurrence characteristics of the response words, we utilize the triplet of three response words from each participant to construct an activation word network for “identity,” as shown in Figure 2. These “response-response” networks, formed by the joint representation of individual perceptions, represent the social representational pattern of “conceptions of identity.” In accordance with the research objectives, this study has constructed four social representational vocabulary networks of “conceptions of identity.”

#### 2.4.2 Network structure analysis

The whole network of social representations of identity perceptions is an activation word network consisting of freely associated response words (nodes) and their co-occurrence relationships (edges). Based on quantitative methods of network analysis (de Nooy et al., 2005; Liu, 2009), the following indicators are adopted to measure the structural characteristics of the social representations of identity perceptions among the new social strata. The meanings and calculation methods of each indicator are as follows:

Firstly, the number of locally connected subnetworks in the undirected social representation network of identity perceptions is calculated as the component index, and then its main components are analyzed. In this study, a component refers to the largest (weakly) connected subnetwork within the social representation network of identity perceptions, while the main component is the largest locally connected subnetwork composed of a subset of response words and the connections among them.

Lastly, from the perspectives of node degree and cohesive subgroups, degree, K-core, and intranuclear participation (Wang et al., 2012) are used as indicators to quantitatively analyze the local structural characteristics of social representations of identity perceptions among the new social strata. “Degree” refers to the number of connections a response word possesses. K-core is the largest subnetwork in a core (or cloud group) where the degree of all response words is no less than K. In this study, the highest-level K-core and its constituent words are regarded as the central core and core elements of the social representations of identity perceptions among the new social strata. Intranuclear participation measures the ratio of the degree of a response word within a K-core to its degree in the entire network. A higher value indicates that the external connections of the response word are limited to within the K-core subnetwork, and an intranuclear participation value of 60% is generally considered the threshold for strong internal participation.

## 3 Results

### 3.1 Identification of the “identity perception” content of the new social strata

**FIGURE 3:**
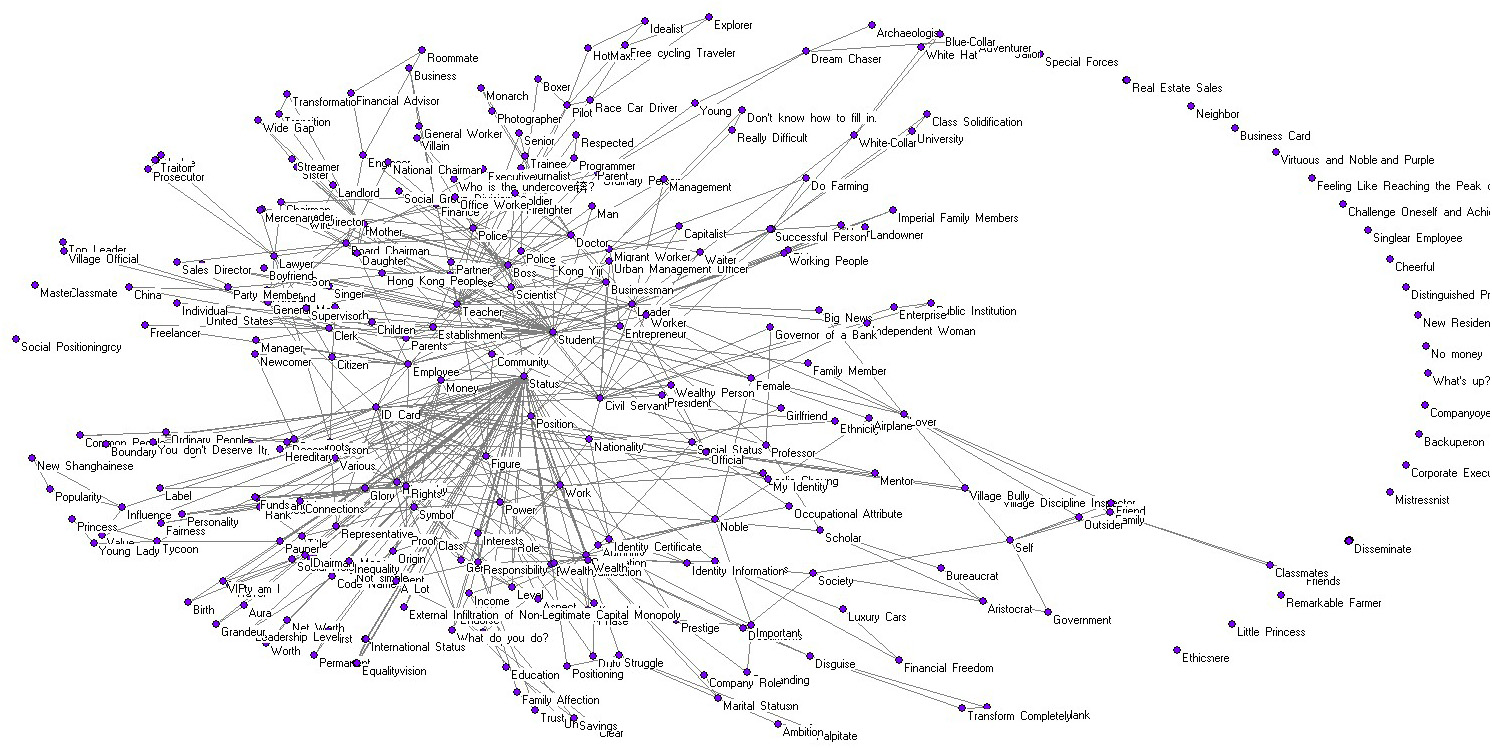
Principal component visualization network graph for the social representation of “identity perception.”

In the social representation network model of the “identity perception” of the new social strata, the scale (size) comprises 368 response words. Through analysis, 30 components with a minimum size of no less than 3 can be extracted, among which the largest component contains 276 words, accounting for 75% of the total scale. The visualization of this network is presented in Figure 3.

### 3.2 Structural analysis of the “identity perspective” of the new social strata

For the largest component, K-core decomposition shows that five relatively dense identity subnets can be solved. In the 2-core network, there are 198 response words, of which “Leader” has the highest nuclear degree and total degree. The 3-core comprises 26 response words, with “Authority” demonstrating the greatest degree centrality. In the 4-core, consisting of 29 response words, “Father” occupies the most central position in terms of nuclear degree and total degree. In the 5-core, “Status” emerges as the most central element. This progression from “Leader” to “Status” across K-core levels reveals a structural hierarchy in the identity representation network.

### 3.3 Core elements of the “identity perspective” of the new social stratum

**FIGURE 4:**
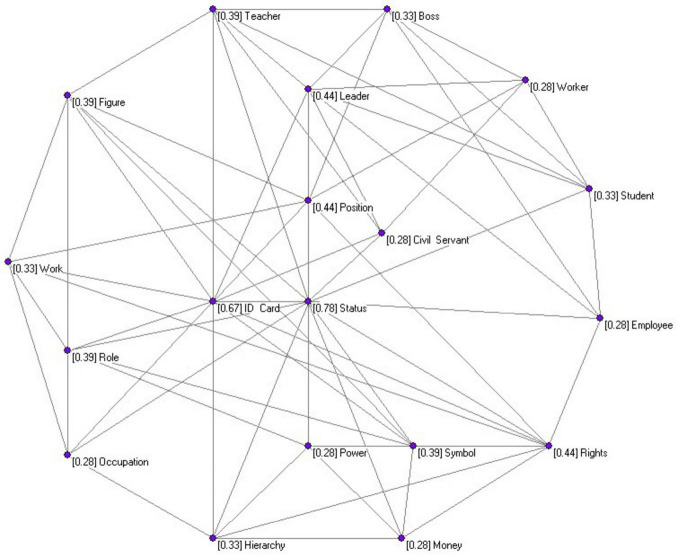
The social representation network diagram of the “central core identity” among the new social strata.

According to the implications of K-core analysis in network metrics, the most condensed subnetwork with the highest K-core level and its constituent elements can be regarded as the central core identity and its core elements in the cognitive representation of the “identity perception” among the new social strata. The K-core analysis outcomes indicate that the 37 core elements and their connection patterns within the highest 4-core subnetwork can be structured into a network model as illustrated in Figure 4.

In the central core identity network, the centrality of the core elements varies significantly, arranged from highest to lowest according to their intra-nuclear degree, as depicted in Table 1.

**TABLE 1:** The core elements of the social representation of the “identity perspective” of the new social stratum.

Core identity	Coreness	Total degree	Core participation percentage
Status	14	83	16.87
ID card	12	30	40.00
Leader	8	23	34.78
Rights	8	16	50.00
Position	8	12	66.67
Figure	7	9	77.78
Teacher	7	28	25.00
Role	7	13	53.85
Symbol	7	11	63.64
Boss	6	22	27.27
Work	6	8	75.00
Hierarchy	6	17	35.29
Student	6	38	15.79
Occupation	5	15	33.33
Power	5	10	50.00
Civil servant	5	19	26.32
Money	5	8	62.50
Worker	5	9	55.56
Employee	5	15	33.33

## 4 Discussion

### 4.1 The hierarchical network structure of the identity perception of the new social strata

Our findings point to a paradox at the core of China’s newest social stratum, where identity is defined as much by the state as it is by self-determination. This dichotomy extends to the ranks and ranks of their own social status categories. Their representation of identity contains abstractions such as status or position at the center, leaving specific occupational identities on the fringe. The tension can then only be fully appreciated against a backdrop that breaks from the more established understanding. In contrast to Western models in which identity is typically profession-specific (Ibarra, 1999), our approach suggests that institutional recognition still prevails over market pressures as institutions work toward professionalization. Our trisection of identity presentation aligns with the way Lamont (2000) sees moral boundaries as dividing members of different social groups according to their values and allowing people to secure some measure of respect, which is articulated by the categorical, relational, and symbolic expressions. As one of the emerging social strata, they need to establish their positionalities at the crossroads of non-socialist sectors without the same types of institutions to rely on as state-sector workers, while still possessing relatively substantial resources. Therefore, it makes sense that the self-representations of these actors might be used as a source of cognitive understanding about how to pursue their social positions, based both on market successes and a claim to cultural legitimacy.

In the socialist market economy system in China, economic liberalization is underway without commensurate political liberalization. References to concepts such as “builders” and “patriots,” even when obtained using neutralized words that would appear to be free from state influence during concept elicitation, are paramount in participants’ identities, revealing how deeply state ideology shapes participants’ self-conceptions, but simultaneously, it is also an identity formed under the sway of marketization as “socialist individualism,” which holds onto both socialist ideology and market competition. The keyword network illustrating lexical associations reflecting people’s social interpretation of identity formation is charted out in Figure 4, and through K-core decomposition, an investigation into society’s agreement on the term is carried out, turning some words among the new social strata and expounding the configuration pattern of the core cognitive elements. From the network structure statistics, a group of core cognitive elements (*N* = 344) constitute the basic configuration, and this configuration is primarily composed of 272 cognitive elements, all of which match the core of the social representation theory, that is, “identity,” forming the way in which people present their identity. The decomposition of the primary subgraph by the K-core produces four well-separated small component networks with different K-core levels, in which each component has one central identity node serving as the core of influence or with global consensus from the society. As the component sub-graphs move from the outer periphery to the center, these identities are “leader,” “authority,” “father,” and “status,” whose salience amplifies due to the higher-order cognitive interactions. The organizational roles of the most salient identity elements contribute to the formation of a multiple and multi-layered hierarchical structure that characterizes the “identity perception” of the new social strata, which is comprised of various cognitive elements. The hierarchical structure described above also bears great resemblance to the morphological structure of a mental lexicon and represents the layer-by-layer hierarchy characteristic of conceptual knowledge representation (Castro and Siew, 2020; Collins and Loftus, 1975). The predominance of “leader” emphasizes the value pursued by the white-collar work class in China’s new social class, which matches with Bourdieu’s (1984) idea on symbolic capital: leadership is a symbol of distinguished position and recognition. As this group rarely possesses official prestige, as do those from the state sector, leadership positions in non-state enterprises allow access to distinction (Li, 2010). And as such, the high centrality of this concept indicates the strong orientation toward rank-based hierarchical advancement instead of horizontal specialization.

Being central to the conception of “Father” as an identity, one cannot overstate the importance of positioning it within a culturally situational context, one that is beyond the occupation to which it pertains. Almost synonymous with filial piety, family perpetuation, and patriarchy as central tenets in Chinese society, Yan (2003) finds that fatherhood serves as an identity to hold the individual at an anchor during drastic social changes for many in the new middle class experiencing geographical movement as well as shifts in family structures. This study indicates that market reform in China has, rather than worked against, increased the centrality of such family identities as a check against prevailing forms of market individualism. Most strikingly, “status” stands out as the major term in every K-core decomposition. In this context of China’s unique societal change, fast-paced growth creates great space for status mobility with intense status anxieties (Zhang, 2019). It is different from the Western class system because the heritage of cultural capital cannot cushion status insecurity. The status telescoping that resulted from China’s compressed modernity was identified by Whyte (2010). This requires constant validation of one’s social standing through demonstrable status markers. For the new stratum group whose institutional positioning is still unclear, they are also driven to pursue a higher status as both an emotional need for security and a strategic way to sail through in this highly complex and uncertain context. On the other hand, authority denotes the authoritative power and influence that makes others place confidence in and observe respect for the entity, indicating an individual, organization, or concept which enjoys the most honor and highest status in its own discipline and can make people feel safe and compliant (Dictionary Editing Office, China Social Sciences Language Institute, 2016). Prestige can be used to indicate the status and influence that individuals or entities hold in a certain range of scope or certain contexts of society and human relationships, reflecting one’s status and role identity, which are inherently socio-dynamic related concepts, and the term “father” essentially denotes male parenthood that involves a role identity in line with society’s consensus regarding paternal responsibilities, with group and other-based affiliation propping this up. The legitimacy to acknowledge it revolves around achieving social rank and recognition, vouching for one’s self-worth and evaluating the meaning of existence by emphasizing one’s attributes. In general, status represents “position, condition or rank in human relationships” (Cihai Editorial Committee, 2009). Psychological status denotes a person’s or a group’s hierarchical standing or position in society or a group, with their rights, privileges, prestige, power relative to others, and degree of societal and group affiliation, based on societal expectations and the personal traits acknowledged by social groups (Lin et al., 2003). Status is a broad term, covering various meanings from factual areas (material spaces) to intangible aspects (roles and identities), but it plays an important role in individual self-conceptions and social identity structures, being an important element in face-to-face social situations. Taking into consideration the view of social representations theory from above, the “identity perception” of the new social stratum presented through the “biographical testimony reports” is expressed as a hierarchical network organization consisting of multiple cognitive units situated across each level in the model, ranging from peripheral levels to central levels, featuring a semiotic gradation moving from particular to general and from singular to plural. This supports the assertion that the social representation of identity underlies hierarchical social networks.

### 4.2 Social consensus on the core identity of the new social strata

Network analysis has unveiled that within the cognitive representation of the “identity concept” among the new social strata, a central-core identity system comprising 37 core elements emerges from their collective experiences and memories. This system is characterized structurally by small-world properties and scale-free attributes, indicating a highly efficient and sparsely connected network. In the identity central-core system, any two identities are related via several steps, based on semantic association or co-occurrence relationships. All of these identities share a converging focus on status, situated at the topmost node of the pyramid structure that organizes identity concepts. The centralizing trend gives status important discursive authority and leads and regulates the way individuals construct their identities. Five central elements—“status,” “student,” “boss,” “employee,” and “occupation”—exhibit low core participation, suggesting they act as organizational hubs, integrating a vast array of identity elements from other levels. In contrast, 17 identity elements, including “official,” “worker,” “human,” “nationality,” “role,” “job,” “money,” “businessman,” “personage,” “position,” “citizen,” “proof,” “symbol,” “representative,” “scientist,” “wealthy individual,” and “entrepreneur,” show high core participation. That means they are less attached to the identity elements of another level. Also, the five identity elements “position,” “figure,” “symbol,” “work,” and “money” are only linked to several elements in the central-core system and do not connect to any identity elements at any other level; this structure stresses that the network of the central-core system is tight and makes it easy for the information about their own identity to be transmitted between others, and indicates that members of the new social strata are in general socially consistent with the new social stratification identity recognition and self-recognition. The categorization, relational, and symbolic dimensions of identity representations emerge in an organic fashion rather than because of our theorizing of them. The triangulation technique did so in three consecutive stages: initially, there was a clear tendency for some of the 37 key components to cluster together in the initial network visualizations (e. g., Berger et al.). Occupation-based terms such as “teacher,” “lawyer,” and “doctor” formed one clearly identifiable cluster. We tested our observation by performing an exploratory clustering analysis with the walktrap algorithm (Pons and Latapy, 2005), revealing three statistically significant clusters (Q = 0.37). Second, we employed reflexive coding; namely, five bilingual researchers coded the 37 elements according to their pure semantic meaning, completely free from translations. In the absence of prior acquaintance with theoretical typologies, their consensus coding (interrater reliability K = 0.82) generated spontaneously three categories that precisely corresponded to the categories of our final typology. Furthermore, we verified these dimensions by comparing them with preliminary qualitative interview data from before the main survey itself; when given an open-ended question on identity importance, participants repeatedly referred to all three dimensions of primacy.

Categorical dimension: “What really counts is what position you hold” (Participant 17).

Relational dimension: “Who you know determines your opportunities” (Participant 42).

Symbolic domain: “People will judge me based on how I dress or what material stuff I own” (Participant 8).

Triangulation testing has confirmed our framework. This confirms that our 3D structural model is empirically grounded, and it lines up with the existing theoretical frameworks on the topic of identity (Stets and Burke, 2000; Lamont and Molnár, 2002). Our categorical level answers the question “What am I?,” and these identities represent what individuals choose to align themselves with based on formal organizational positions and their professional credentials. The national dimension reflects the positional identities found in the various social strata, their answers to “where do I stand?,” which involves comparative status, power differentials and network positions. The symbolic dimension comprises the material and cultural representations answered by “how am I perceived?,” which center around visible indicators of status and belonging, so as to become the means through which new social strata identify themselves, describe, and interpret identity. Categorical identities denote the differentiation and understanding between the status of social people located within the various categories of an occupational, familial or national relationship. Relational identities are determined based on positions taken by individuals in various social networks associated with different class statuses, roles, positions, and statuses, accompanied by different kinds of rights and power. Symbolic identity represents how individuals in the emerging middle-class evaluate the material media that social actors use to identify and symbolize self and essence. Notably, “ID Card” was the second most central element (coreness = 12) in the identity representation network. In China, an ID card is a unique marker for status, privileges, and relationships, since individual identity can be legally grounded and therefore traceable, attributable, and trackable through ID cards. Documents represent very important ways of showing that we belong to a society and also serve as recognition of our legal rights. In China, documents such as an ID card or Hukou document can show not only people’s official documents for getting services but also the way that shows the representative of their official identity (Wang and Liu, 2001). For example, for these new types of society, where some have moved around the country and gained different occupations. Documents materialize and legitimate contested claims to social recognition and institutional inclusion. By focusing on the ID card, we show that such institutional mechanisms of identification shape people’s subjectively constructed identities today in contemporary China. Thus, for the new social strata, in its broadest sense, “ID Card” must be conceived as having categorical, relational, and symbolic dimensions that structure a common perception among people regarding the core elements of the social subjects that make up the new social strata. Put more simply: the categorization of different subjects, as well as their status as distinct social positions, is exemplified through specific socialized symbolizations of subject groups.

### 4.3 Limitations and future directions

This article has some restrictions that deserve notice. Firstly, convenience sampling restricts the possibility for the generality of the Chinese social strata that the findings can be extrapolated to the overall population. Future research will try to employ a stratified random sampling approach within different provinces and professions. Secondly, due to the selection of politically laden stimulus items, subjects’ social desirability response may influence their answers. These tasks might lead people to coordinate their responses with mainstream discourses rather than being true to their own immediate identity experience. Later research can use less biased stimulating materials or multimodal methods (e.g., in-depth interviews) to check results. Furthermore, the cross-sectional design does not allow for investigating the change of different types of identity representations in reaction to changes in people’s socioeconomic status, and longitudinal studies that can track these shifting identities before and after major life transition points will offer important complementary insights. Lastly, examining cognitive associations using methods such as word-elicitation tasks might exclude emotional and embodied aspects of identity. Including qualitative techniques into analysis might shed light on the way people live their identity beyond representations.

## Data Availability

The original contributions presented in this study are included in this article/supplementary material, further inquiries can be directed to the corresponding authors.
